# Laforin, a Dual Specificity Phosphatase Involved in Lafora Disease, Is Present Mainly as Monomeric Form with Full Phosphatase Activity

**DOI:** 10.1371/journal.pone.0024040

**Published:** 2011-08-26

**Authors:** Vikas V. Dukhande, Devin M. Rogers, Carlos Romá-Mateo, Jordi Donderis, Alberto Marina, Adam O. Taylor, Pascual Sanz, Matthew S. Gentry

**Affiliations:** 1 Department of Molecular and Cellular Biochemistry and Center for Structural Biology, College of Medicine, University of Kentucky, Lexington, Kentucky, United States of America; 2 Instituto de Biomedicina de Valencia, CSIC and Centro de Investigación Biomédica en Red de Enfermedades Raras (CIBERER), Valencia, Spain; Laurentian University, Canada

## Abstract

Lafora Disease (LD) is a fatal neurodegenerative epileptic disorder that presents as a neurological deterioration with the accumulation of insoluble, intracellular, hyperphosphorylated carbohydrates called Lafora bodies (LBs). LD is caused by mutations in either the gene encoding laforin or malin. Laforin contains a dual specificity phosphatase domain and a carbohydrate-binding module, and is a member of the recently described family of glucan phosphatases. In the current study, we investigated the functional and physiological relevance of laforin dimerization. We purified recombinant human laforin and subjected the monomer and dimer fractions to denaturing gel electrophoresis, mass spectrometry, phosphatase assays, protein-protein interaction assays, and glucan binding assays. Our results demonstrate that laforin prevalently exists as a monomer with a small dimer fraction both *in vitro* and *in vivo*. Of mechanistic importance, laforin monomer and dimer possess equal phosphatase activity, and they both associate with malin and bind glucans to a similar extent. However, we found differences between the two states' ability to interact simultaneously with malin and carbohydrates. Furthermore, we tested other members of the glucan phosphatase family. Cumulatively, our data suggest that laforin monomer is the dominant form of the protein and that it contains phosphatase activity.

## Introduction

Laforin is a dual specificity phosphatase encoded by the *EPM2A* (epilepsy of progressive myoclonus type 2 A) gene [1], [2]. Autosomal recessive mutations in *EPM2A* cause Lafora disease (LD) [1], [2]. LD is a type of myoclonic epilepsy where the patient undergoes neurodegeneration and severe cognitive decline. Occurrence of seizures begins in the second decade of patient's life and LD results in fatality within ten years of the first seizure [3], [4], [5], [6]. Pathognomonic insoluble intracellular carbohydrate/glucan deposits termed Lafora bodies (LBs) are observed in brain, skeletal muscle, skin, liver, and other tissues of LD patients [6].

Laforin contains an amino-terminal carbohydrate-binding module (CBM) belonging to the CBM20 family and a carboxy-terminal dual specificity phosphatase (DSP) domain [1], [2], [7], [8]. Recombinant laforin hydrolyzes phosphotyrosine and phospho-serine/threonine substrates *in vitro* [7], [9], but laforin is structurally most similar to non-proteinaceous DSPs [10]. Studies from our group and others demonstrate that laforin is a unique phosphatase in that it binds and dephosphorylates phospho-glucans [11], [12], [13], [14]. Supporting this finding is the fact that LBs contain increased amounts of phosphate compared to normal glycogen [12], [15]. A recent report finds that phosphate is incorporated into glycogen by glycogen synthase as an error during synthesis [16]. Laforin was also shown to dephosphorylate glycogen synthase kinase-3β (GSK3β) [17], [18], [19]; however, ourselves and other groups have not observed dephosphorylation of GSK3β by laforin [11], [12].

Autosomal recessive mutations in the *EPM2B* gene that encodes the protein malin also cause LD [20]. We previously demonstrated that malin is a single-subunit E3 ubiquitin ligase that binds and ubiquitinates laforin [21]. In addition, multiple labs demonstrated that laforin functions as a scaffolding protein for the laforin-malin complex-mediated down regulation of proteins involved in glycogen metabolism, such as protein targeting to glycogen (PTG), amylo-1,6-glucosidase,4-alpha-glucanotransferase (AGL/GDE), and muscle glycogen synthase (GS) [22], [23], [24], [25]. However, these results are currently in dispute given recent results generated from a malin knockout mouse [26], [27].

A recent study reported that laforin forms SDS-resistant dimers both *in vitro* and *in vivo* [28]. Surprisingly, it was reported that laforin dimers possess the vast majority of laforin phosphatase activity and that monomeric laforin is nearly inactive [28]. Many proteins undergo self-association to form dimers and oligomers and this dimerization gives them structural and functional advantages [29]. Among phosphatases, dimerization is commonly observed in receptor protein-tyrosine phosphatases (RPTPs) [30], [31]. Homodimerization of RPTPα-1, CD45, and SAP-1 was demonstrated to inhibit their activity [31], [32], [33], whereas dimerization of RPTPσ affected its ligand binding [34]. However, dimerization of non-receptor PTPs such as that observed in alkaline phosphatase, bovine protein tyrosine phosphatase, and vaccinia virus H1 is a rare phenomenon [35], [36], [37], [38], [39].

Laforin is a cytoplasmic phosphatase and therefore the occurrence of laforin dimerization is both intriguing and applicable in determining the molecular etiology of Lafora disease. One could envision dimerization affecting glucan-binding, protein-protein interactions, and/or phosphatase activity of laforin. In addition, laforin oligomerization could be involved in the formation of LBs. Proteinaceous accumulations are a common theme in neurological disorders. Even though LBs are mainly made up of insoluble glucans, unlike the protein deposits seen in Alzheimer's and Parkinson's disease, it has been suggested that LD pathology may have a component linked to mis-regulation of the proteasome [40], [41]. However, structural and functional consequences of laforin dimerization have not been fully elucidated. Thus, we aimed to define the effect of dimerization on the function of laforin and its possible role in the etiology of Lafora disease.

## Methods

### Plasmids and protein purification

pET21a Hs-laforin-HIS_6_, pET21a At-SEX4-HIS_6_(Δ81), pET21a Cm-laforin-HIS_6_, pCDNA3.1NF-malin, and pET-GST-malin-HIS_6_ are described in refs [13], [21]. pGEX6P1-laforin was obtained by digesting the corresponding pEG202-laforin plasmid [25] with BamHI/SalI and subcloning the fragments into pGEX6P1 (GE Healthcare). Other plasmids used in this study were pCMVmyc-laforin [25] and pGEX4T1-VHR, a generous gift of Dr. Rafael Pulido (Centro de Investigacion Principe Felipe, Valencia, Spain). Dr. Marcelo Sousa generously provided purified arnA protein. Recombinant proteins were purified from soluble bacterial lysates in buffer (50 mM Tris pH 8.0, 300 mM NaCl, 0.5% Triton X-100, complete mini protease inhibitor (Roche)) using Ni-NTA resin (Qiagen) followed by gel filtration chromatography using an AKTA Purifier with a HiLoad 16/60 Superdex 75 or a Superdex 200 10/300 GL size exclusion column (GE Healthcare) as previously described [42].

### Protein gel electrophoresis and mass spectrometry

Denatured gel electrophoresis was carried out using NuPAGE 10% Bis-Tris gels. Coomassie-stained bands were cut from gels, digested with trypsin, desalted, and analyzed by MALDI TOF/TOF. The peptides were searched with Protein Pilot against Swiss-Prot database. The mass spectrometric analysis was performed at the University of Kentucky, Center for Structural Biology Protein Core Facility.

### Phosphatase activity measurements

*para*-nitrophenylphosphate (pNPP, 50 mM)/3-O-methyl fluorescein phosphate (OMFP, 0.5 mM) and amylopectin (0.9 µg/µl) were used to determine phosphatase activity of Hs-laforin, Cm-laforin, and SEX4. Assays were performed as previously described [13]. Briefly, reactions were carried out in buffer containing 0.1 M sodium acetate, 0.05 M bis-Tris, 0.05 M Tris-HCl, 2 mM dithiothreitol, pH 6.0 for pNPP assay or in buffer (0.1 M Tris-HCl pH 8, 40 mM NaCl, 2 mM DTT) for OMFP assay. The absorbance of the product was measured at 410 nm for pNPP assay and at 490 nM for OMFP assay. Phosphatase activity using amylopectin and malachite green reagent was measured at 620 nm as described previously [11]. The reaction used 45 µg of amylopectin as a substrate and the same buffer as used in pNPP assay. The reaction was terminated by addition of 0.1 M *N*-ethylmaleimide prior to the addition of malachite green reagent.

### Cell culture and immunodetection

Human embryonic kidney (HEK293) cells were grown in DMEM (Lonza) supplemented with 100 units/ml penicillin, 100 µg/ml streptomycin, 2 mM glutamine, 10% inactivated fetal bovine serum (GIBCO). 1.5×10^6^ cells were plated onto 60 mm culture dishes the day before transfection. Cells were transfected with 1 µg of pCMVmyc-laforin plasmid using Lipofectamine 2000 (Invitrogen). Twenty-four hours after transfection, cells were scraped on ice in lysis buffer [10 mM TrisHCl pH 8; 150 mM NaCl, 15 mM EDTA; 0.6 M sucrose, 0.5% nonidet P-40 (NP-40), protease inhibitor cocktail (Roche), 1 mM PMSF, 50 mM NaF and 5 mM Na_2_P_2_O_7_]. Cells were lysed by repeated passage through a 25-gauge needle. To analyze proteins under non-reducing conditions, cell extracts (25 µg) were diluted in SDS- and DTT-free loading buffer (125 mM Tris-HCl, 20% glycerol) and analyzed by regular SDS-PAGE and immunoblotting using an anti-myc (Sigma) antibody.

### Glucan-binding assay

Glucan-binding assays were performed as previously described [7]. Briefly, laforin monomer and dimer, normalized to laforin content, were incubated with amylopectin (5 mg) suspended in 0.5 ml of buffer containing 50 mM Tris, 150 mM NaCl, pH 7.5 for an hour at 4° C. Co-sedimentation with amylopectin was measured by centrifuging the samples at 106000×g for 1.5 hrs and analyzing the supernatant and pellet fractions thus obtained by immunoblotting with anti-HIS_6_ antibody. For testing the effect of malin on glucan-binding ability of laforin, purified HIS_6_-GST-malin was added to laforin monomer and dimer along with amylopectin and same method was followed with use of mouse monoclonal antibody to detect laforin (Abnova).

### Immunoprecipitations

HEK293 cells were transfected with FLAG-tagged malin using Lipofectamine 2000 (Invitrogen) as above. Cells were lysed using modified RIPA buffer (Tris pH 8.0 50 mM, NaCl 150 mM, NP40 1%, glycerol 10%, NaF 10 mM, and EDTA 0.4 mM). FLAG-malin was immunoprecipitated with anti-FLAG M2 agarose beads (Sigma), the beads were washed twice with modified RIPA buffer, and then incubated with laforin monomer or dimer for 1 hr at 4° C. Following this incubation, the beads were washed once with modified RIPA buffer and proteins were eluted with 50 µl of 2× NuPage sample buffer (Invitrogen) at 95°C. Western analysis was used to detect laforin with a rabbit polyclonal antibody (GeneTex) and an anti-FLAG-HRP antibody (Sigma) to detect malin.

### Statistical analysis

Values are given as means ± SEM of at least three independent experiments. Differences between groups are analyzed by two-tailed student's t-tests or by one-way analysis of variances. The significance has been considered at * p<0.05 and ** p<0.001, as indicated in each case. Data for protein purification, gel electrophoresis, glucan-binding, and immunoprecipitation is representative of al least three independent determinations.

## Results

### Monomeric laforin is the dominant species

**Figure 1:**
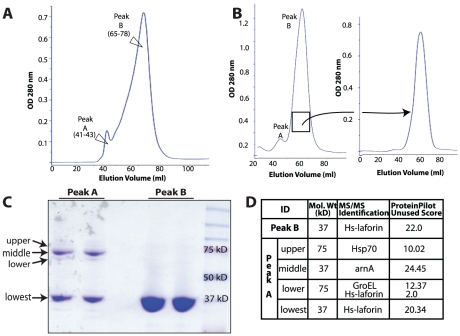
Laforin monomer is abundant compared with its dimer form. (A) The chromatogram is of Hs-laforin-HIS_6_ purified using a Superdex-75 column and contains two distinct peaks, peak A and peak B. This is a representative of 6 purifications. (B) Fractions from peak B (65–78) were collected, concentrated, and re-loaded onto a Superdex-75 column. The chromatograms are representatives of 4 experiments. (C) Proteins from these peak A (41–43) and B (65–78) were collected, separated using denaturing gel electrophoresis, and stained with Coomassie. (D) The gel bands of monomeric and dimeric peaks were excised, trypsin digested, and subjected to mass spectrometric identification (MS/MS).

As previously reported by Liu et al. [28], size exclusion chromatography of purified Hs-laforin-HIS_6_ expressed in bacteria results in two prominent peaks, laforin dimer (peak A) and laforin monomer (peak B) (Figure 1A). To evaluate if we had fully resolved these peaks, we collected the fractions from peak B in Figure 1A, combined the fractions, concentrated them, and passed over the same size exclusion column. As expected, peak B produced a single peak (Figure 1B). These results demonstrate that the two peaks are distinct and that the monomer fraction does not convert into a dimer.

Resolving the constituents of these peaks by gel electrophoresis under fully denaturing conditions revealed that the monomeric peak (peak B) is highly pure and present at the expected 37 kDa band. However, the dimeric peak (peak A) showed a single band at 37 kDa and three defined bands around 75 kDa (Figure 1C). To determine the identity of the proteins, we performed mass spectrometry on each band. In the peak B fraction, laforin was the only protein identified. As expected under denatured conditions, peak A also contained laforin at 37 kDa. However, peak A also contained the *E. coli* proteins arnA, Hsp70, and GroEL as well as laforin at around 75 kDa, of which arnA was the predominant band (Figure 1D). Although only a fraction of the lower band in the 75 kDa range was laforin, we were surprised to find some dimeric laforin resistant to fully reduced and denatured conditions. However, the majority of dimeric laforin is converted to a monomer when subjected to high levels of reducing agent, SDS, and boiling.

Our mass spectrometry results identified arnA as the major contaminant in the dimeric fraction A (Figure 1). arnA is a bi-functional polymyxin-resistant protein that catalyzes oxidative decarboxylation of UDP-Glucuronic acid and formylation of UDP-Ara4N [43]. The Coomassie-stained gel and mass spectrometry results demonstrate that approximately 50% of the proteins in fraction A are not laforin. A previous report suggested that the laforin dimer was fully resistant to denaturation by heat and SDS treatment [28]. However, our results demonstrate that the major band in the 75 kDa range is *E. coli* arnA, and that the vast majority of laforin in peak A is denatured and runs as a 37 kDa species. Importantly, these results confirm that laforin does form dimers, but only a minor fraction of recombinant laforin dimerizes. In addition, the dimer fraction (peak A) is contaminated with *E. coli* proteins.

### Laforin monomer and dimer have equal phosphatase activity

**Figure 2:**
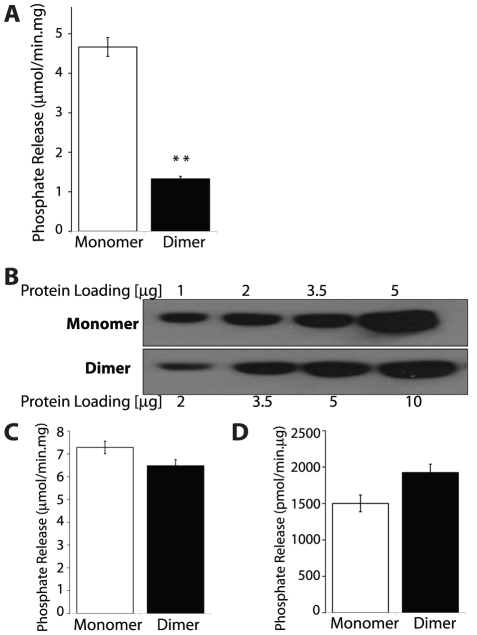
Laforin monomer and dimer have equal phosphatase activity. (**A**) Specific activity of laforin monomer (peak B) and dimer (peak A) fractions obtained by size exclusion chromatography (Figure 1A) against pNPP. The activities are compared based on total protein content. (**B**) A representative immunoblotting image of varying concentration of laforin monomer and dimer fractions detected using anti-HIS_6_ monoclonal antibody. (**C**) Specific activity for laforin monomer and dimer fractions against pNPP. The activities are compared based on total laforin content from the blot in panel B. (**D**) Phosphate release measured by malachite green assays using amylopectin as a substrate for laforin monomer and dimer fractions. Normalization of the activity was carried out for laforin content. All values are means ± SEM (**p<0.001) analyzed by independent sample ‘t’ test.

A previous study reported that monomeric laforin lacks phosphatase activity and that only laforin dimers possesses phosphatase activity [28]. In order to determine the effect of laforin dimerization on its phosphatase activity we utilized two assays. First, we employed the exogenous substrate para-nitrophenylphosphate (pNPP) to test the phosphatase activity of laforin monomer and dimer. When normalized to total protein content, laforin monomer (peak B in Figure 1A) displayed three times higher pNPP phosphatase activity compared to laforin dimer (peak A in Figure 1A) (Figure 2A). This finding is in contrast to the previous report that dimeric laforin has a higher specific activity than monomeric laforin [44]. However, we demonstrated in Figure 1 that the laforin dimer fraction is contaminated with multiple *E. coli* proteins, and thus phosphatase activity normalized for total protein content does not truly represent the phosphatase activity of the dimer form.

To account for the *E. coli* proteins in the dimer fraction, we normalized both monomer and dimer fractions for laforin content using a laforin-specific antibody and Western analysis (Figure 2B). We determined the total protein concentration for the monomer and dimer fractions, loaded different amounts of each fraction (1–5 µg for the monomer and 2–10 µg for the dimer), separated the proteins by SDS-PAGE, Western transferred the proteins, and probed the blots with an α-HIS_6_ antibody. We found that the amount of laforin in the dimeric fraction was decreased by 50% as compared to the monomeric fraction (Figure 2B). Therefore, 4 µg of total protein from the dimer fraction only contains 2 µg of dimeric laforin.

Given the *E. coli* contamination in the dimer fraction (peak A), one cannot simply use total protein as a means to assess the amount of laforin in the dimer fraction. Therefore, we normalized the dimer fraction so that we analyzed equal amounts of laforin in the pNPP assay and all subsequent assays. After adjusting for total laforin amount instead of total protein, we found that the pNPP phosphatase activity for the laforin monomer and dimer fractions was equal (Figure 2C). To ascertain that these findings were not confounded by phosphatase activity that arnA might possess, we tested and confirmed that arnA lacks *in vitro* phosphatase activity as measured by the pNPP assay.

pNPP is a good exogenous substrate to monitor phosphatase activity, but it does not share structural similarities with glycogen, a biological substrate of laforin [11], [12]. It could be possible that laforin monomer and dimer differ in their glycogen phosphatase activity even though their pNPP activity is equal. To test the glucan phosphatase activity of laforin, we utilized a malachite green assay where molybdate in malachite green forms a complex with inorganic phosphate released from a phospho-substrate and this causes a colorimetric change. Amylopectin is a phosphorylated glucan that resembles glycogen and is a suitable substrate to measure glucan phosphatase activity [11]. When normalized for laforin levels, malachite green assays demonstrated that both monomer and dimer forms of laforin have equal glucan phosphatase activity (Figure 2D). Therefore, in contrast to a previous report, our results demonstrate that both monomeric and dimeric laforin are equally capable of removing phosphate from an exogenous substrate and from phospho-glucans.

### Oligomerization of glucan phosphatases from other Kingdoms

Kingdom Plantae/Archaeplastida genomes lack a true ortholog of laforin, but their genomes do encode for a functional equivalent of laforin [13]. The *Starch EXcess4* gene encodes the SEX4 protein in *Arabidopsis thaliana* [45], [46]. SEX4 possesses a chloroplast-Targeting Peptide (cTP), a DSP domain, and a CBM [46], [47]. We previously demonstrated that SEX4 is a glucan phosphatase and human laforin can partially complement mutations in *SEX4* [13]. While plants lack a true laforin ortholog, laforin is conserved in the genome of five protozoans [48]. The most distantly related laforin ortholog is in the red alga *Cyanidioschyzon merolae*, Cm-laforin, which is 25% identical to human laforin [13]. We previously demonstrated that Cm-laforin possesses the same biochemical signature as laforin, in that they both bind glucans and can dephosphorylate phospho-glucans [13]. Given the functional similarities between human laforin, Cm-laforin, and SEX4, we used SEX4 and Cm-laforin in the current study to evaluate if the oligomerization phenomenon is true of all glucan phosphatases.

**Figure 3:**
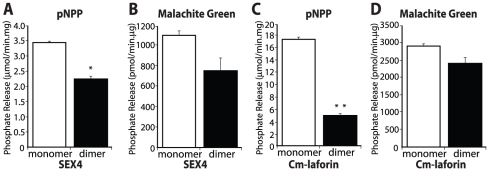
Phosphatase activity of monomeric and dimeric forms of SEX4 and Cm-laforin. (A) Specific activity of SEX4 monomer and dimer fractions obtained by size exclusion chromatography (Figure S1) against pNPP. The activities are compared based on total SEX4 content (Figure S2). (B) Phosphate release measured by malachite green assays using amylopectin as a substrate for SEX4 monomer and dimer fractions. The activities are compared based on total SEX4 content. (C) Specific activity for Cm-laforin monomer and dimer fractions obtained by size exclusion chromatography (Figure S1) against pNPP. The activities are compared based on total Cm-laforin content (Figure S2). (D) Phosphate release measured by malachite green assays using amylopectin as a substrate for Cm-laforin monomer and dimer fractions. The activities are compared based on total Cm-laforin content. All values are means ± SEM (*p<0.05, **p<0.001) analyzed by one-way ANOVA.

We purified SEX4 and Cm-laforin using a similar two-step purification method that included size exclusion chromatography. For each protein, we observed that multiple peaks were eluted from the column, similar as we observed for human laforin (Figure S1). We analyzed the ratio of SEX4 and Cm-laforin monomer and dimer fractions via immunoblotting (Figure S2), as was performed for Hs-laforin in Figure 2B. Then we tested the phosphatase activity of SEX4 using both pNPP and malachite green assays. We found that monomeric SEX4 has a slightly higher specific activity than dimer using both pNPP and malachite green as substrates (Figure 3A and 3B). For Cm-laforin, the specific activity against pNPP of the monomer form was three times higher than that of dimer; whereas the malachite green assay showed that the glucan phosphatase activity of Cm-laforin was similar for monomer and dimer (Figure 3C and 3D).

Hs-laforin, SEX4, and Cm-laforin all belong to glucan phosphatase family [10]. These results demonstrate that glucan phosphatases from different Kingdoms are all active in the monomeric form. Interestingly, dimeric forms of glucan phosphatases from different Kingdoms exhibit different phosphatase activity, suggesting different modes of actions for each dimeric form.

### Laforin dimerization is affected by redox conditions

**Figure 4:**
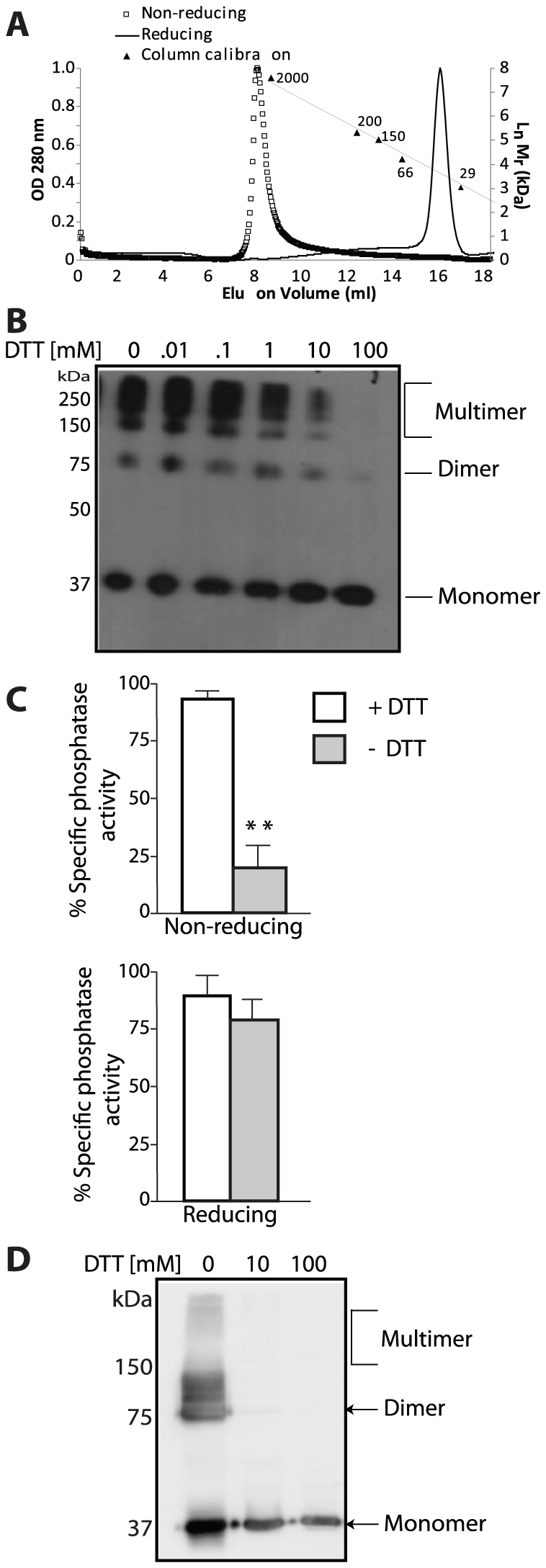
Reducing agents prevent laforin oligomer formation. (A) Gel-filtration analysis on Superdex 200 10/300 GL column of human laforin stored in the presence or absence of reducing agents (10 mM DTT). A laforin sample stored at −20°C in the absence of DTT showed an elution profile (non-reducing peak; squares) corresponding to an apparent molecular weight higher than 2,000 kDa. A laforin sample stored in the presence of 10 mM DTT (reducing peak; line) showed an elution profile corresponding to an apparent molecular weight of approximately 37 kDa. Calibration of the column with size standards is indicated; ordinates indicate the natural logarithm (Ln) of molecular weight (Mr) in kDa. (B) The non-reducing peak of purified laforin was treated or not with different amounts of DTT before analysis by non-reducing gel electrophoresis and immunoblotting using anti-laforin antibodies. The position of the monomeric, dimeric and multimeric forms of laforin is indicated. (C) Phosphatase activity of the non-reducing and reducing peaks of laforin was measured in the presence or absence of 10 mM DTT in the reaction mixture. All values are means ± SEM (**p<0.01; n: 3) analyzed by independent sample ‘t’ test. (D) Cell extracts from HEK293 cells transfected with plasmid pCMVmyc-laforin were analyzed by non-reducing gel electrophoresis. When indicated, samples were treated with different amounts of DTT before loading them into the electrophoresis gel. The position of the monomeric, dimeric and multimeric forms of myc-laforin is indicated.

Given the difference between our data and a previous report suggesting that laforin monomer is inactive, we decided to determine a possible cause for this discrepancy. We found an interesting result when laforin was stored at −20°C in the absence of reducing agent. We purified monomeric laforin using Ni-NTA resin and size-exclusion chromatography by collecting peak B (Figure 1A), and then stored the purified protein at −20°C in the presence or absence of a reducing agent (10 mM DTT). These purified proteins were then reloaded onto an analytical size-exclusion column (Superdex 200) and, in the case of proteins stored in the absence of DTT we observed multimeric species that separated as high molecular weight proteins (larger than 2,000 kDa) (Figure 4A; non-reducing peak). However, proteins stored in the presence of a reducing agent eluted as a single peak around 37 kDa (Figure 4A; reducing peak). Thus, monomeric laforin again remains as a monomer and does not convert into dimeric laforin similar to our findings in Figure 1B.

The finding that storage of laforin in low levels of DTT is necessary prompted us to further examine the effect of reducing agents on laforin oligomerization and phosphatase activity. When we analyzed the non-reducing peak of laforin (Figure 4A) by gel electrophoresis under non-reducing conditions (no SDS and no DTT was present in the sample loading buffer and the samples were not heated), we observed the presence of laforin monomers, dimers, and multimers (Figure 4B, first lane). However, if we added increasing amounts of DTT we found that laforin oligomerization was reversed and at 100 mM DTT only monomeric laforin remained (Figure 4B). These results suggest that laforin oligomerization is very sensitive to oxidation, and that multiple species of laforin form under non-reducing conditions. These species may result from intermolecular disulphide bond formation among the nine cysteine residues present in laforin. Additionally, these results show that the amount of DTT commonly utilized in phosphatase assays (1–2 mM DTT) does not affect dimerization or multimerization. However, these low levels of DTT are necessary to keep the catalytic cysteine reduced [49].

Dual specificity phosphatases employ a two-step catalytic mechanism. After nucleophilic attack of the substrate phosphorus atom, a phosphoryl-cysteine intermediate is formed before hydrolysis of the intermediate and release of phosphate [49], [50], [51], [52]. In oxidative environments, this catalytic cysteine is modified and inactivated [53]. In order to define the relationship between oxidation of laforin and its phosphatase activity, we examined the phosphatase activity of laforin that was purified and stored in the absence of DTT (non-reducing peak, Figure 4A) using the exogenous substrate 3-O-methyl fluorescein phosphate (OMFP). We found that the phosphatase activity of laforin was dependent on the presence of DTT in the reaction buffer: without DTT the activity was abolished, whereas in the presence of 10 mM DTT the activity was significantly higher (Figure 4C). Alternatively, a laforin sample purified and stored in the presence of DTT (reducing peak, Figure 4A) was fully active, even in the absence of DTT in the phosphatase reaction buffer (Figure 4C). These results demonstrate that the phosphatase activity of monomeric and dimeric laforin is both dependent on a reduced environment.

In order to further probe the effect of reducing conditions on laforin dimerization, we analyzed the oligomeric status of laforin in mammalian HEK293 cells lysed in the presence and absence of reducing agent. When the cell extracts were prepared in the absence of DTT, clear monomeric, dimeric, and multimeric species were resolved by non-reducing gel electrophoresis (Figure 4D). However, if cells were lysed in the presence of 10 mM DTT (or higher concentrations) only monomeric laforin was detected. These results suggest that redox conditions may regulate laforin dimerization and that cellular oxidative stress may affect laforin oligomerization. It is important to note that these results strongly suggest that no laforin dimer is present with ≥10 mM DTT. The phosphatase assays performed in the presence of DTT (+ DTT, Figure 4C) employed 10 mM DTT in the assay buffer. Therefore, all of the laforin present should be in monomeric form and the monomeric laforin does possess phosphatase activity, supportive of our findings in Figure 2.

### Laforin dimerization does not affect glucan binding

**Figure 5:**
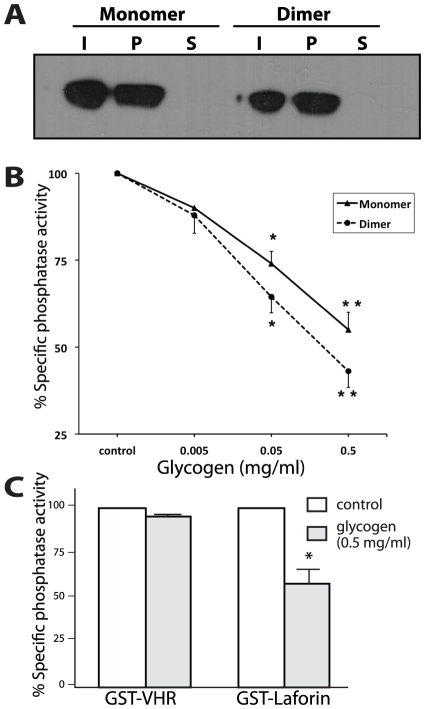
Dimerization of laforin does not affect its ability to bind glucans. (A) Equal amounts of monomeric (0.5 µg peak B fraction, Figure 1A) and dimeric (1 µg peak A fraction, Figure 1A) laforin were incubated with amylopectin and glucan-binding assay was performed as described in Methods. A representative image of the I (input), P (pellet), and S (supernatant) fractions analyzed by Western blotting is presented. (B) The OMFP phosphatase activity of GST-laforin fusion protein purified from bacteria was measured in the presence of different amounts of glycogen in the reaction mixture. We assigned the maximal phosphatase activity in the absence of glycogen as 100% and then compared activity in the presence of glycogen to this maximal amount. (C) Phosphatase activity of GST-laforin and GST-VHR in the absence or presence of glycogen (0.5 mg/ml) in the reaction mixture. As in Figure B, we assigned maximal phosphatase activities as 100% and compared activities in the presence of glycogen to the untreated samples (control). Values are means ± SEM of three independent experiments (*p<0.05) analyzed by independent sample ‘t’ test.

The dimer interface of laforin could involve the carbohydrate-binding module (CBM), and if so dimerization could provide a mechanism to modulate glucan-binding. To test whether dimerization of laforin impacts its ability to bind glucans, we utilized a glucan-binding assay. In this assay, proteins are added to an amylopectin solution and the mixture undergoes ultracentrifugation. Proteins in the pellet and supernatant fractions are then assessed by Western analysis. Proteins with glucan-binding ability are retained in the pellet fraction and proteins lacking this ability are observed in the supernatant fraction. Immunoblotting of the pellet and supernatant fractions from the glucan-binding assay showed that both laforin monomer and dimer bind to amylopectin and are enriched in the pellet fraction (Figure 5A). Therefore, dimerization of laforin does not inhibit its glucan-binding.

Multiple groups have reported that glycogen inhibits laforin phosphatase activity [54], [55], [56]. In agreement with these results, we observed a clear inhibition of monomeric laforin phosphatase activity in response to higher levels of glycogen in the reaction mixture (Figure 5B). Similarly, we found that glycogen inhibits the phosphatase activity of dimeric laforin (Figure 5B). As expected, glycogen did not affect the phosphatase activity of VHR (Figure 5C), a dual specificity phosphatase that lacks a CBM. Therefore, glycogen inhibits the phosphatase activity of monomeric and dimeric laforin.

### Laforin dimerization does not affect its association to malin but the presence of malin differentially affects glucan-binding of monomer and dimer

**Figure 6:**
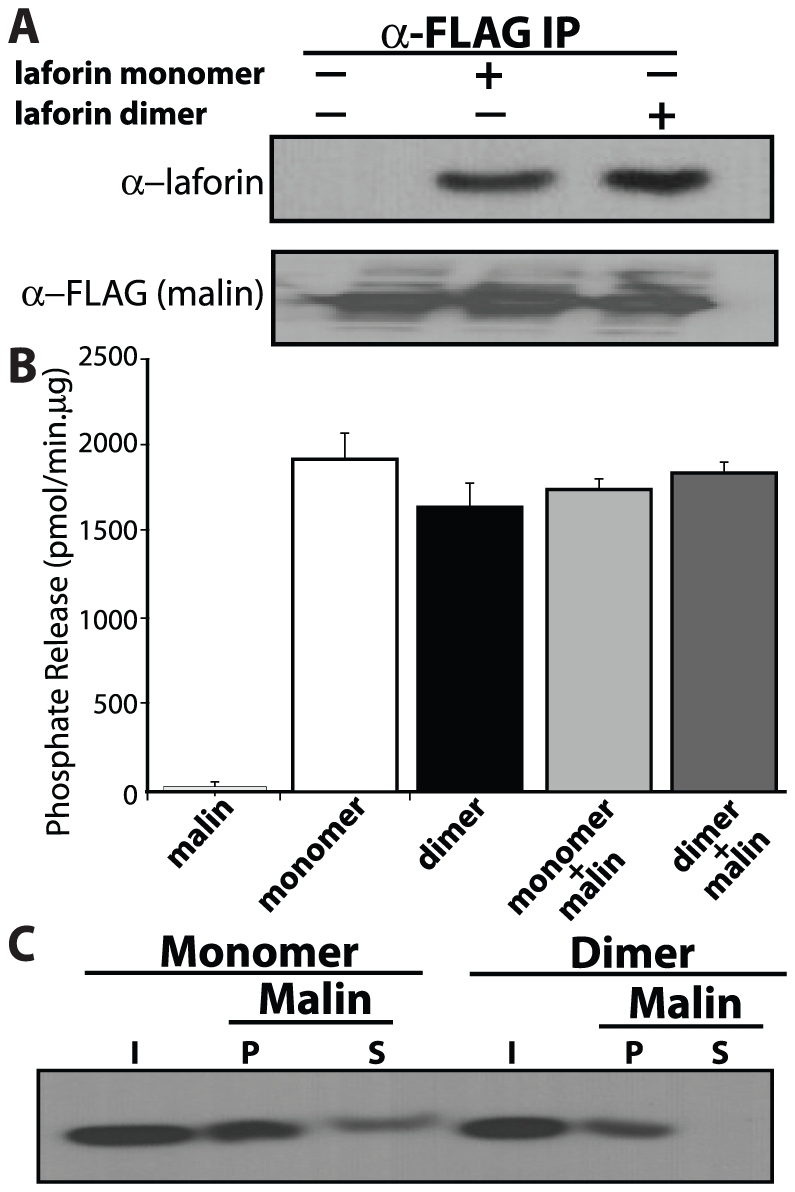
Dimerization of laforin does not affect its ability to associate with malin. (A) Equal amount of monomeric and dimeric laforin used in section A of Figure 6, were mixed with FLAG-malin and immunoprecipitation was carried out as described in Methods. A representative image showing detection of monomeric and dimeric laforin in samples immunoprecipitated using anti-Flag agarose beads is presented. (B) Phosphatase activity of monomeric and dimeric laforin in the presence of malin was determined using amylopectin as substrate. (C) Representative image demonstrating the presence in the I (input), P (pellet), and S (supernatant) fractions from the glucan-binding assay of laforin monomer (0.5 µg) and dimer (1.0 µg), that had been previously mixed with Hs-malin-HIS_6_ (1.0 µg). The membrane was blotted with α-laforin antibody.

As discussed in the introduction, laforin forms a functional complex by associating with malin and this complex is involved in ubiquitination and proteasomal degradation of multiple proteins involved in glycogen metabolism [21], [22], [24], [25]. The differences in the structure of monomeric and dimeric laforin could alter its ability to interact with malin that could change the scaffolding function of laforin. Therefore, we investigated the ability of malin to interact with monomeric or dimeric laforin by co-immunoprecipitation. We transfected HEK293 cells with FLAG-tagged malin, lysed the cells, immunoprecipitated FLAG-malin with anti-FLAG M2 agarose beads, and washed the beads multiple times. We then incubated the bound FLAG-malin with monomeric or dimeric laforin, again washed the beads, eluted bound proteins with NuPage sample buffer, and analyzed the proteins by Western analysis. We observed that malin interacted with both monomeric and dimeric laforin to equal degrees (Figure 6A). Thus, laforin dimerization does not impair its interaction with malin.

Given that malin and laforin form a complex, we decided to test the activity of monomeric and dimeric laforin in the presence of malin using the malachite green assay. The presence of malin did not affect the phosphatase activity of either monomeric or dimeric laforin (Figure 6B). Thus, both forms maintain phosphatase activity in the presence of malin.

Although there was no change in the ability of monomeric and dimeric laforin to bind malin, we surmised that the binding of malin with laforin could have a differential impact on glucan-binding. To determine how monomeric and dimeric laforin interact with glucans in the presence of malin, we incubated equal amounts of GST-malin-HIS_6_ with both forms of laforin and then performed the glucan-binding assay as described above. The presence of malin decreased the binding of monomeric laforin to glucans as indicated by the presence of monomeric laforin in both the pellet and supernatant fractions (Figure 6C). However, the presence of malin only minimally decreased the binding of dimeric laforin to amylopectin (Figure 6C). Thus, the ability of dimeric laforin to bind glucans is not impaired by malin. These data suggest that monomeric laforin binds to malin and that the laforin-malin complex does not bind as tightly to glucans as laforin monomer alone.

## Discussion

Defining laforin dimerization is necessary to evaluate the functional and pathological role of laforin in Lafora disease. In the present study, we demonstrate that monomeric laforin is the most abundant form of the phosphatase under normal reduced conditions. Our study also establishes that laforin phosphatase activity is similar for both monomer and dimer species. In addition, monomeric and dimeric laforin exhibit equal ability to associate with malin and bind glucans. However, monomeric laforin has decreased glucan-binding capacity in the presence of malin, while the glucan binding of dimeric laforin is not affected by malin. Another key finding of this study is that oxidative conditions play a key role in both the phosphatase activity and oligomerization of laforin. These results demonstrate that lack of a reducing agent drives laforin oligomerization and abolishes the phosphatase activity of laforin. Conversely, the presence of glycogen did not impact laforin oligomerization, but glycogen did decrease its phosphatase activity. Cumulatively, our data establish that monomeric and dimeric laforin possess similar phosphatase activity, glucan binding, and dimerization is enhanced by increased oxidation and that glucan binding in the presence of malin is decreased for monomeric laforin and not for the dimer.

Most primary papers and reviews on Lafora disease have formulated hypotheses with the assumption that mutations inactivating monomeric laforin give rise to Lafora disease. However, a recent study reported that monomeric laforin lacks phosphatase activity [28]. Thus, one of the key reasons to initiate this work was to determine if monomeric laforin possesses phosphatase activity, and if not then to re-assess our understanding of disease mutations. We utilized multiple lines of evidence to definitively demonstrate monomeric laforin is an active phosphatase: **1)** monomeric laforin purified via Ni-NTA resin and resolved using size exclusion chromatography possesses pNPP activity (Fig. 2C); **2)** the same monomeric laforin also possesses activity against the phospho-glucans, a biologically relevant substrate (Fig. 2D); **3)** monomeric SEX4 from *Arabidopsis* possesses phosphatase activity against pNPP and a phospho-glucan (Fig. 3A and B); **4)** monomeric laforin from the red algae *C. merolae* possesses phosphatase activity against pNPP and a phospho-glucan (Fig. 3C and D); and **5)** when DTT levels are increased to ≥10 mM DTT only monomeric laforin exists (Fig. 4D) and Figure 4C demonstrates that laforin is fully active under these conditions. The above results clearly demonstrate that monomeric laforin is the most abundant form of laforin and that it contains full phosphatase activity. The lack of phosphatase activity of monomeric laforin reported by Liu et al. [44] is possibly due to the absence of reducing agents either during purification and/or storage.

Hs-laforin, Cm-laforin, and SEX4 all contain a CBM and DSP domain and all belong to the newly discovered class of glucan phosphatases [10], [11], [13]. To define how dimerization affects other glucan phosphatases, we purified both Cm-laforin and SEX4 and tested their pNPP and glucan phosphatase activity. Similar to Hs-laforin, SEX4 and Cm-laforin both formed dimers. Contrary to what we observed for Hs-laforin, the phosphatase activity of the monomeric SEX4 and Cm-laforin was higher than the dimeric form. These data indicate that glucan phosphatases are functional in their monomeric state, but that differences are present across Kingdoms.

Our results from cell culture suggest that laforin dimerization may be a dynamic process. The sensitivity of the oligomeric-monomeric transition to the presence of reducing agents indicates that inter-molecular Cys-Cys bridges play a key role in oligomer formation. These data suggests that laforin is present *in vivo* as a combination of monomeric and oligomeric forms, and changes in the cellular reducing conditions may regulate the transition from one state to the other. In support of this hypothesis, a recent paper found that a laforin mutation, laforin-Ser25Ala, is unable to interact with itself in both a yeast two-hybrid system and in mammalian cell culture experiments [57].

We found that both monomeric and dimeric laforin bind glucans with equal affinity. This finding suggests that sites involved in laforin dimerization do not affect the conformation of essential CBM residues involved in glucan-binding. Next, we analyzed the inhibitory role of glycogen on laforin phosphatase activity. We observed that this inhibitory role is not due to alterations in the oligomeric-monomeric transition, as the presence of glycogen did not affect oligomer formation. Moreover, a dual specificity phosphatase lacking a carbohydrate-binding domain (VHR) was resistant to glycogen inhibition. Therefore, our results suggest that glycogen either induces a conformational change in laforin structure or inhibits phosphatase activity because it blocks the entry of substrates to the phosphatase catalytic site.

In addition to phosphatase activity, we analyzed laforin monomer and dimer for their ability to interact with malin. The equal association of monomeric and dimeric laforin with malin suggests that both forms possess active scaffolding function and form laforin-malin complexes. Phosphatase activity of laforin monomer and dimer was not affected by presence of malin, which suggests that laforin-malin complex is functionally active. While we did not observe a difference in glucan-binding between the two forms, we did observe a difference when malin was present. Malin affected the glucan-binding of only monomeric laforin and did not affect glucan-binding of the dimer form.

In conclusion, our findings establish that monomeric laforin is an active enzyme, and that laforin dimerization is not essential for its physiological activity. In addition, we found that monomer and dimer possess equal specific activity in removing phosphate from both generic and biologically relevant substrates. Our *in vitro* results and previous *in vivo* data clearly demonstrate that monomeric laforin is far more abundant than the dimer and that changes in the cellular reducing conditions may regulate the transition from one state to the other. These results are especially germane in terms of defining the form(s) of laforin that is most relevant to the etiology of Lafora disease.

## Supporting Information

Figure S1 **Purification of** ***Arabidopsis*** **Starch EXcess4 (SEX4) and** ***C. merolae*** **laforin (Cm-laforin).** (A) The 32 kDa SEX4 protein was purified in a similar manner as Hs-laforin. The chromatogram is of SEX4-HIS_6_ purified using a Superdex-75 and contains three distinct peaks. This is a representative of 5 purifications. (B) The 74 kDa Cm-laforin protein was purified in a similar manner as Hs-laforin. The chromatogram is of Cm-laforin-HIS_6_ purified using a Superdex-200 and contains three distinct peaks. This is a representative of 4 purifications.
(EPS)

Figure S2 **Quantification of monomer versus dimer of SEX4 and Cm-laforin.** (A) A representative immunoblotting image of varying concentration of SEX4 monomer and dimer fractions detected using anti-HIS_6_ monoclonal antibody. (B) A representative immunoblotting image of varying concentration of Cm-laforin monomer and dimer fractions detected using anti-HIS_6_ monoclonal antibody.
(EPS)
